# A material stress test study on occurrence of leakage and material failure of peritoneal dialysis (PD) catheters

**DOI:** 10.1038/s41598-021-89643-0

**Published:** 2021-05-13

**Authors:** Matthias Klingele, Martin Carstens, Lea Baerens, Matthias W. Laschke, Wolfgang Metzger, Danilo Fliser, Clemens M. Meier

**Affiliations:** 1 Department of Nephrology, Hochtaunuskliniken, Zeppelinstrasse 20, 61352 Bad Homburg, Germany; 2 Department of Internal Medicine, Nephrology and Hypertension, Saarland University Medical Centre, Homburg, Saar Germany; 3 Department of General, Visceral, Vascular and Pediatric Surgery, Saarland University Medical Centre, Homburg, Saar Germany; 4 Institute for Clinical and Experimental Surgery, Saarland University Medical Centre, Homburg, Saar Germany; 5 Department of Trauma, Hand and Reconstructive Surgery, Saarland University Medical Centre, Homburg, Saar Germany

**Keywords:** Preventive medicine, Peritoneal dialysis

## Abstract

Peritonitis is a common complication of peritoneal dialysis (PD). Our root cause analysis allowed to attribute some cases to leakage of the PD catheter. Accordingly, a clinically based stress test study on potential material damage issues of PD catheters was performed, focusing on material damage caused by cleaning, de- and attachment procedures during dialysate changes and on the individual storage methods of PD catheters between dialysate changes. PD catheters were exposed to both chemical stress by repeating dialysate-flow and physical stress simulating de- and connecting, fixation, pressure, flexing, folding etc.—simulating standard clinical daily routine of 8–10 years PD catheter usage. Potentially by normal usage caused damages should be then detected by intraluminal pressure, light- and electron microscopy. The multi-step visual control showed no obvious damages on PD catheters nor any leakage or barrier indulgence. Our tests simulating daily routine usage of PD catheters for several years could not detect any material defects under chemical or physical stress. Hence, we presume that most PD catheter damages, as identified cause for peritonitis in some of our patients, may be due to accidental, unnoticed external damage (e.g. through scissors, while changing dressings) or neglecting PD catheter handling specifications.

## Introduction

Peritoneal dialysis (PD) is a common renal replacement therapy. Overall, PD is used in Germany with 5–6%, in Spain with 10%, in Australia with 20% and over in Hongkong with 70% of all dialysis patient^1,2^.

Peritonitis is a dreaded complication of PD as it leads to significant death rate^3,4^. Due to technical improvements and innovations the rate of peritonitis dropped significantly; currently 0,5 episode every year is still considered acceptable by nowadays standards^5,6^. Our study focusses on the question how to further lower the risk of peritonitis through possibly avoidable PD catheter defects.

PD patients are well trained in handling and hygienic standards. Nevertheless, many cases of peritonitis originate from e.g. insufficient hygienics. All the more, we could not instantly explain some cases of peritonitis in role model patients showing excellent and very experienced perfect handling and hygienic skills. Moreover, other causes of peritonitis e.g. abdominal infections of other organs or bacterial translocation from the gut due to ischemia had been excluded. Therefore, a material examination of the PD catheter indicated the most likely source of peritoneal infections of namely little leakages. Generally, leakage of PD catheters is a rarely noted complication in literature. However, so far described reasons for the leakage of PD catheters, such as rupture^7,8^, chemical applications^9^ or manufacturing errors^10^, could not be confirmed in our cases. Rather neither we nor the patients could identify any handling and/or storage issues. Following, the systematic research on potential sources of—firstly—unexplainable cases of peritonitis in our patients and the indeed identified leakages of the PD catheter initiated our material stress test idea in a laboratory setting. Only a simulation of the relevant and typical clinical settings allowed to validate purely material tiredness under usage stress while excluding potential accidental and unnoticed external damage when in usage with the patient.

The aim of this study was to evaluate the resistivity of PD catheters under fully realistically clinical routine conditions, but in a laboratory setting to exclude even unnoticed handling mistakes as root cause for material failure issues resulting in peritonitis.

## Methods

A clinically based stress test study on potential material failure issues of PD catheters was conducted. The focus laid on potential material tiredness through normal usage stress such as cleaning and de- and attachment procedures of tubes and adaptors when changing dialysate as well as the individual method of storage of the catheter itself between dialysate changes.

We developed a simulation of the relevant and typical clinical settings, allowing to validate purely material performance or potential tiredness/failure under chemical stress through dialysate and physical stress through de- and connecting, fixation, pressure, flexing, folding etc..

Replicating the clinical routine through laboratory material stress, PD catheters of different production batches were selected. We used in total 10 Tenkhoff Peritoneal Catheters, curled with two cuffs. 8 × Argyle™ Curl Cath Peritoneal Dialysis Catheter (Covidien, Mansfield, MA, USA) and 2 CC12 S 1 H3 of Joline (Joline Gmbh & Co KG, Hechingen, Germany). All catheters are comparable curled tubing with numerous inflow/outflow holes and made of translucent, medical-grade silicone rubber. They all were exposed to a regular chemical and / or physical daily usage. This laboratory material stress test was run for more than three months, effectively simulating up to 8–10 years of operational material stress when

- passing through of commonly applied chemical substances
- physical handling within clinical routine.

The aspect of material tiring purely over calendar time (e.g. chemical material disruption) was excluded due to neither any indication of such issues in the overall clinical study center material experience with medical silicone catheters—nor the possibility of a truly realistic time simulation.

**Figure 1:**
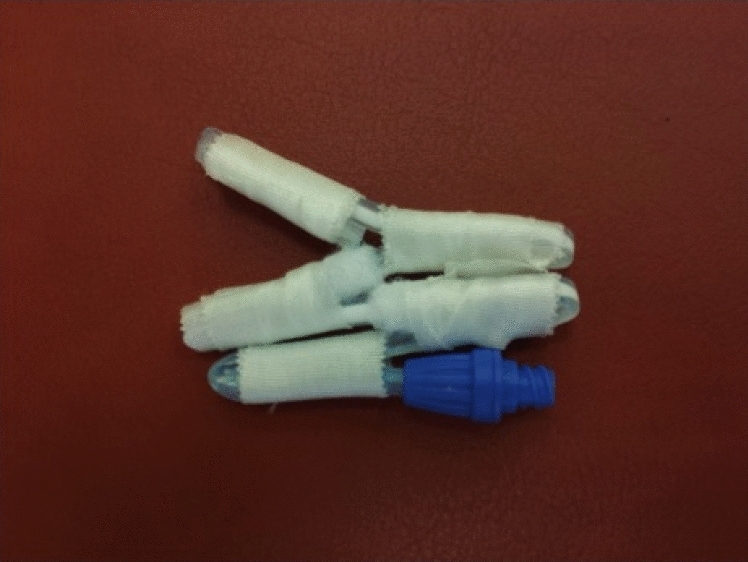
Static physical stress through multiple folding.

Within the laboratory material stress test simulation, the chemical exposure was tested by filling PD catheters with standard dialysate solution (2.3% Fresenius, Fresenius Medical Care, Bad Homburg, Germany) for up to 100 days; renewal of solution every 3–5 days. All applications, e.g. sealing, were either original or of common clinical use. Physical set up when filled was either hanging vertically or laying horizontally. Before and after refill a set of evaluation procedures, e.g. applying different technical methods to measure potential liquid outlet, were performed and documented. Further new and already chemical stress tested PD catheters were then put under static physical stress by multiple folding up (as shown in Fig. 1) and as such stored tightly folded for 11 days.

**Figure 2:**
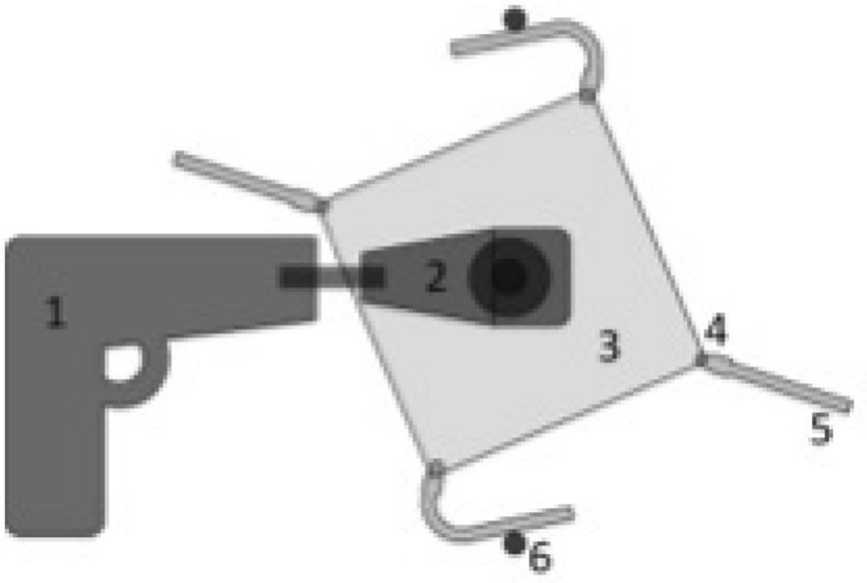
Schematic display of dynamic physical stress test set up. Fixed battery loaded drilling machine (1) drives over an axis and an angle torsional moment (2) a squared birch tree wooden panel. At each square corner 50 mm long PD catheter pieces (5) equipped with Luer-Lock-adapters (4) are attached. Within each full rotation of the wooden panel, each PD catheter piece is twice bend at 100° at the opposite metal sticks (6).

New and already chemical stress tested PD catheters were put under dynamic physical stress by running the PD catheters against a metal piece, making it bend and expand again for 40,000 or even 80,000 times in the exact same spot, as shown in Fig. 2.

PD catheters are often folded and fixed on the abdominal skin. Tight clothes even enhance the pressure on folded catheters. This was simulated within our static physical stress test. In this test, the act of folding and fixing the folded catheter was done extremely, trying to provoque potential material failure within a short time period.

Within our dynamic stress test we tried to simulate repeated mechanical folding and defolding of PD-catheters during changes of dialysate. We calculated 5 changes as mean number of dialysate changes per day, resulting in about 2000 foldings and defoldings per year. Since we could not include aging processes of PD-catheters, we doubled the number of dynamic stress events. Thereby we resulted in 40,000 events of folding and defolding to simulate about 10 years of usage. Although technique survival of peritoneal dialysis in a patient does rarely reach 10 years, we wanted to examinate material failure for this period. Thereby we can assume, that our results would be of interest for a large majority of PD-patients.

**Figure 3:**
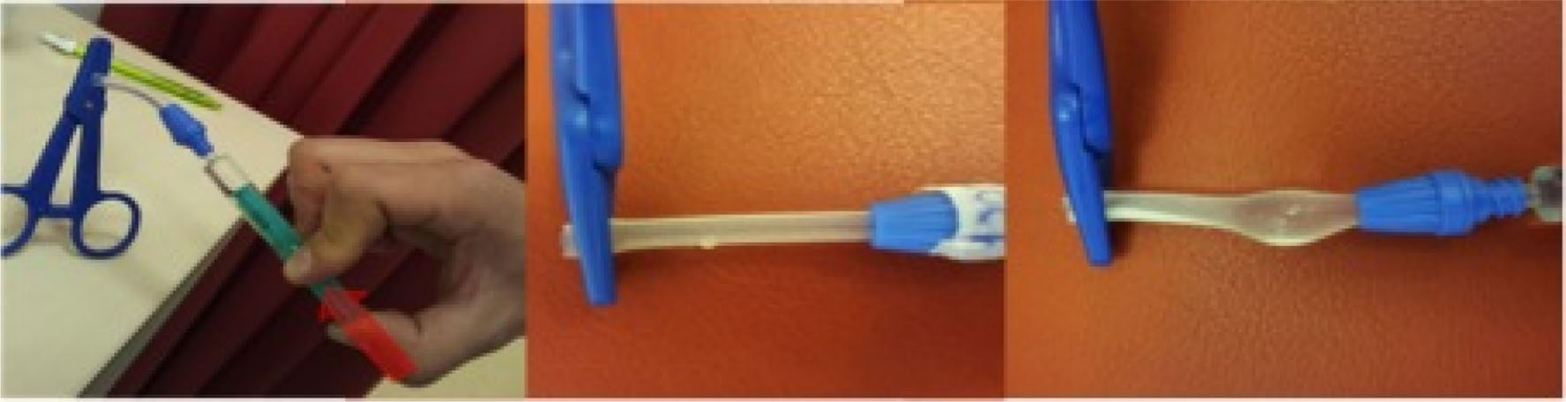
Intraluminal pressure exposure (left) to identify leakages such as e.g. liquid outlets (middle) and/or bulging (right).

All stress-tested PD catheters were evaluated as follows:

- firstly, visual control for obvious damages;
- secondly, intraluminal pressure exposure to identify leakages, such as e.g. liquid outlets and/or barrier leakage as shown in Fig. 3
- thirdly, light microscopy (Leica M651, Leica Microsystems, Wetzlar, Germany) with and without Methylen-blue staining (10%, Art.-Nr.: 457250-1, VWR, Darmstadt, Germany);
- finally, scanning electron microscopic assessments

All results have been systematically documented and evaluated.

## Results

**Figure 4:**
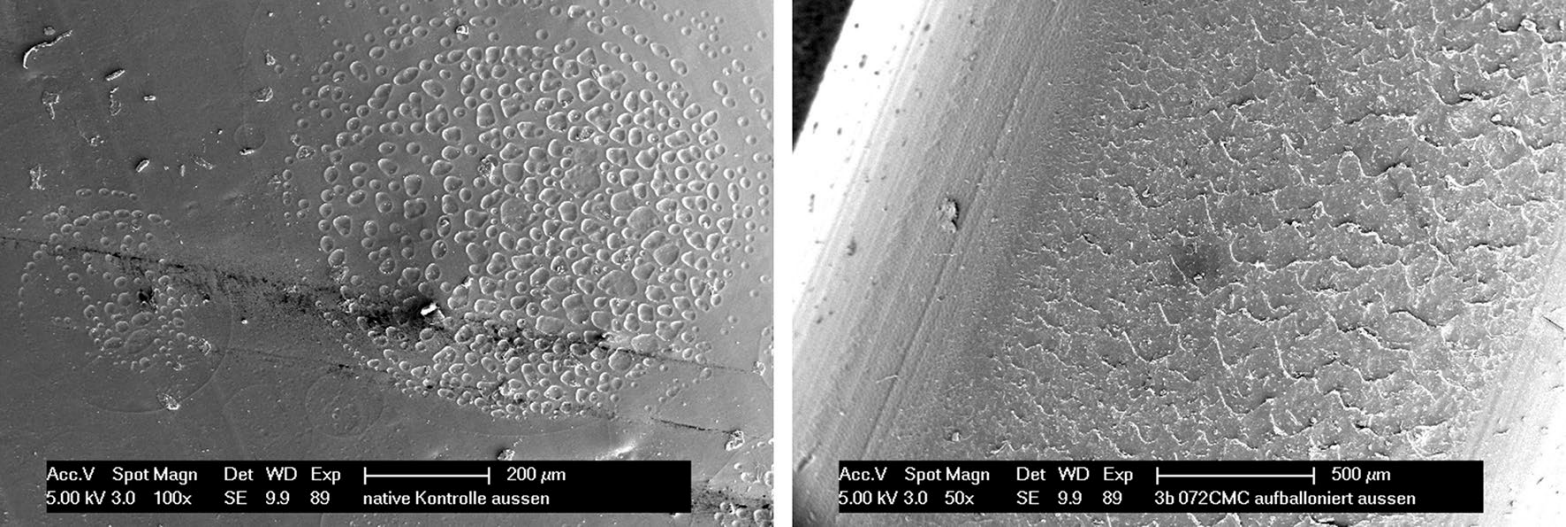
Scanning electron microscopic assessment shows cornfield pattern at the inner surface (left) and scale structure on the outside (right).

The evaluation of all fast motion stress-tested PD catheters through normal clinical usage simulation showed clear findings:

- Firstly, visual control showed no obvious damages, meaning no fractures, no signs of severe material tiredness or material anomalies, no material defects.
- Secondly, intraluminal pressure exposure to identify leakages, such as e.g. liquid outlets and/or barrier permeability as firstly: no detectable material defects.
- Thirdly, light microscopy before and after colouring with Methylen-blue (10%): no visible damage of any wall of any catheter could be displayed. There were only smaller deposits of dirt (dust) on the outside of some catheters detectable.
- Finally, scanning electron microscopy was performed. Here slightest lesions at the very surface on the in- and outside of the catheter wall could be detected. Overall, on the in- and outside of the catheters some deposits (e.g. glucose and mineral salts) could be identified. Moreover, several catheters showed two different kinds of patterns on the surfaces: cornfield pattern or scale structure as shown in Fig. 4. Both were identified after chemical and or physical stress. In any case, only very small longitudinal cracks on the inner and outer surfaces could be detected. None of all lesions was deep enough to allow liquids or air to pass through, nor had a counter lesion on the opposite side—meaning same spot inverse at the in- or outside.

Side note, one untested, by random sample chosen, completely new PD catheter showed slight lesions on the outside, without signs of permeance.

## Discussion

This is the first comprehensive and independent research study of its kind on pure material testing of PD catheters under laboratory, but fully realistically simulated clinical routine conditions while intentionally excluding potential accidental and unnoticed external damage when in usage with the patient. Thereby, no indication at all was found that chemical and/or physical stress as during regular clinical usage would lead to any relevant damage to the PD catheter.

Historically, the access for peritoneal dialyses started with a surgical trocar, developed to rubber catheters, to more soft, polyvinyl intraperitoneal tubes, polyethylene and nylon catheters, and finally silicone catheters with a polyester cuff in 1968, introduced by Tenckhoff^11^. Silicone had shown less local tissue irritation compared to other catheter materials. In addition, silicone is rather resistant, e.g. against resolvents, such as ethanol, which was shown for example in catheters for hemodialysis^12,13^.

Leakage or rupture of PD catheters is rarely described in literature to cause outflow failure or infection. For example, a drainage failure of a silicone double-cuffed straight Tenckhoff catheter due to a break of the PD catheter in the subcutaneous area in a single patient has been reported by Kim^7^. In some cases, chemical applications seem to play a role for rupture of PD catheters^9^. This hypothesis is supported by Khandelwal et al. They describe structural alterations in 6.6% of examined PD catheters in patients using mupirocin at the exit site^14^. Similar results are reported for the use of gentamycin at the exit site resulting in erosions of the silicone material of the peritoneal dialysis catheter^15^. In one report, a PD catheter rupture was caused by mechanical stress through the patient's stretching exercise program^16^. Moreover, spontaneous ruptures of PD catheters are described for more than 25 years^8,17,18^, however, this seems to be a rare problem and the causes are different. In another case, manufacturing error escaping quality control checks could be responsible for rupture of PD catheters^7^.

The hypothesis, that PD catheter-related complications may at times be due to material and/or manufacturing errors, is supported by material research on silicone catheter incidents within hemodialysis: Weijmer et al. reported a spontaneous HD catheter rupture; electron microscopy and X-ray spectral analysis suggested a shortcoming within the production process and/or an undetected production error to have led to the described spontaneous rupture^10^.

Within our PD catheter material testing and examination thereafter, there was no indication for any malfunction, neither indicating a production error nor due to a real stress simulation such as folding repetitively or statically. None of the fast motion stress tested PD catheters simulating normal clinical usage of 8–10 years showed any detectable defect of relevance contraindicating or jeopardizing their unproblematic use. The chemical and physical exposure seem to provoke slight structural signs on the inner surface, but without influence on PD catheter closeness or quality at any time. No leakage or close to leakage spot was found at all, even not under electron microscopic examination. The stress test study could not find any sign of relevant PD catheter material tiredness nor quality issues.

Based on these results, any PD catheter defect when in patient usage and showing an evident leakage, up to visible to the naked eye whole, assumingly originating from incidents beyond standard handling. With some patients, scissors might have touched the PD catheter accidently when e.g. changing dressings. With other patients the potential source could not clearly be identified; it is likely that items of clothing with metal could have damaged the PD catheter during storage at the body.

## Limitations

Our results are not applicable to any PD catheter but only the tested types as we tested only a small number of different types and of different enterprises. Although our simulations aim to cover a quite long time period of normal PD catheter usage, our tests do not cover any material defect aspects beyond the tested stress. Further, we could not apply the stress tests with older or even already used, but so far intact catheters. Therefore, our study gives no information about material failure due to long-term material fatigue. Chemical stress was limited to commonly used PD-solutions. We did not test external chemical stress due to applications of medications e.g. mupirocin and gentamicin or desinfectant solutions and others. This aspect is described in literature. Our study therefore provides no information, whether those chemical stress factors in addition to our physical stress tests would have resulted in material failure.

## Conclusions

**Figure 5:**
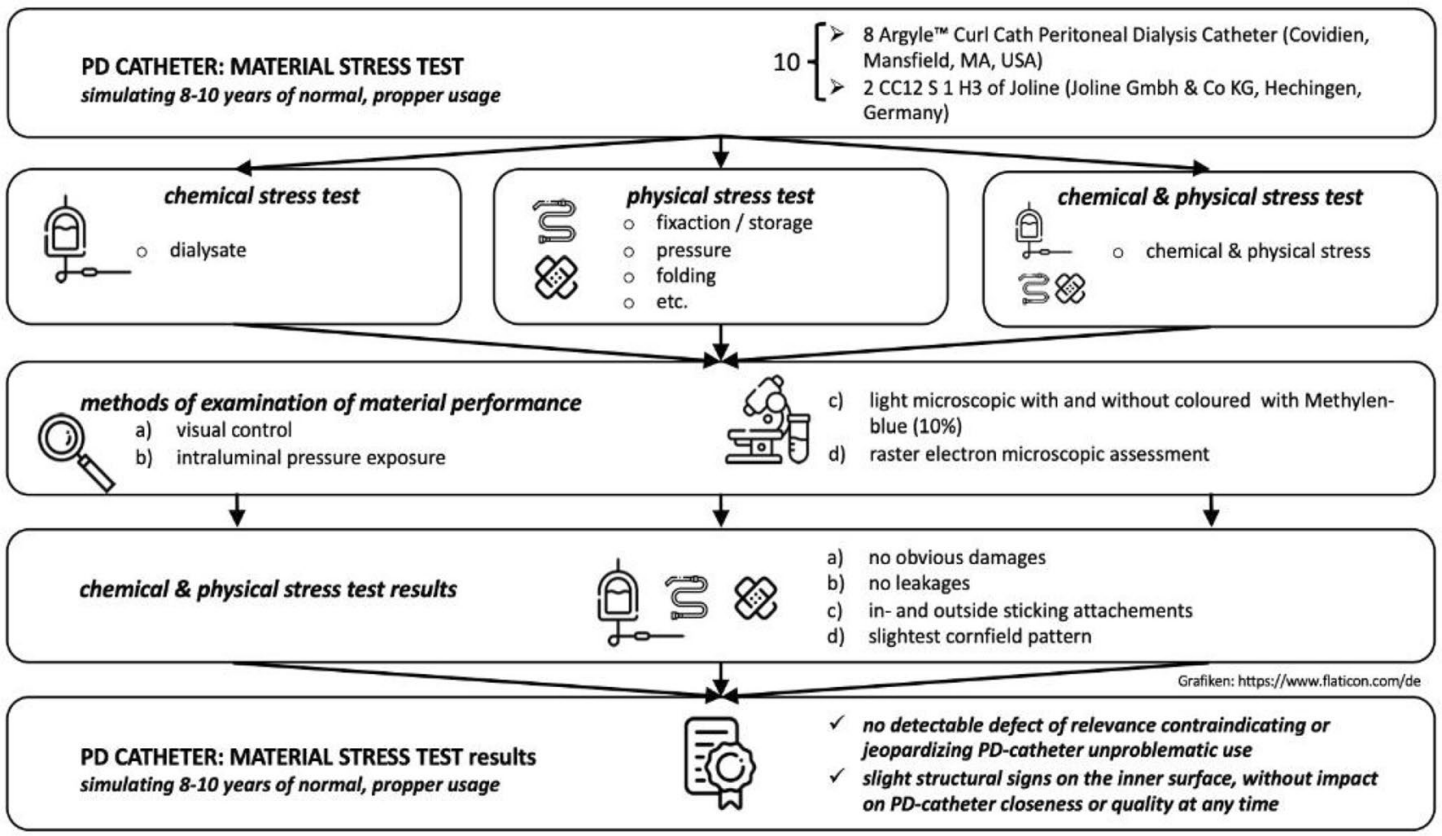
Graphical abstract.

The material stress test study of PD catheters validates the quality of all actually tested batches and pieces. Neither chemical nor physical stress simulation of normal handling of up to 8–10 years led to catheter leakages as shown in Fig. 5. Hence, we have to assume that reported small leakages most likely trace back to handling or application errors, even if unnoticed. However, it is a matter of speculation whether, for example, pointed objects during dressing changes or contact with metal elements led to the leakage. Nevertheless, as most patients denied any handling mistakes in our overall examined peritonitis cases and most small leakages are hardly visible to the naked eye within clinical routine, we started a regular, systematical quality control. Surprisingly, no material malfunction of the PD catheter has been detected since then.

## References

[1] Kwong VW-K, Li P-T (2015). Peritoneal dialysis in Asia. Kidney Dis..

[2] Li PK (2017). Changes in the worldwide epidemiology of peritoneal dialysis. Nat. Rev. Nephrol..

[3] Perl J (2020). Peritoneal dialysis-related infection rates and outcomes: results from the peritoneal dialysis outcomes and practice patterns study (PDOPPS). Am. J. Kidney Dis..

[4] Ye H (2017). The impact of peritoneal dialysis-related peritonitis on mortality in peritoneal dialysis patients. BMC Nephrol..

[5] Li PKT (2016). ISPD peritonitis recommendations: 2016 Update on prevention and treatment. Perit. Dial. Int..

[6] Szeto C-C (2017). ISPD Catheter related infection recommendations: 2017 update. Perit. Dial. Int..

[7] Kim HR (2013). Drainage failure because of spontaneous fracture of the peritoneal dialysis catheter. Perit. Dial. Int..

[8] Wadhawan A, Saxena R (2019). Spontaneous fracture in intramural and intra-abdominal segments of peritoneal dialysis catheters presenting as outflow failure. Perit. Dial. Int..

[9] Riu S, Ruiz CG, Martinez-Vea A, Peralta C, Oliver JA (1998). Spontaneous rupture of polyurethane peritoneal catheter. A possible deleterious effect of mupirocin ointment. Nephrol. Dial. Transplant..

[10] Weijmer MC, Kars SM, ter Wee PM (2001). A scanning electron microscopy analysis of a spontaneous hemodialysis catheter fracture. Am. J. Kidney Dis..

[11] Twardowski ZJ (2006). History of peritoneal access development. Int. J. Artif. Organs.

[12] Landry DL (2015). Effects of prolonged ethanol lock exposure to carbothane- and silicone-based hemodialysis catheters: A 26-week study. J. Vasc. Access..

[13] Guenu S (2007). Mass spectrometry and scanning electron microscopy study of silicone tunneled dialysis catheter integrity after an exposure of 15 days to 60% ethanol solution. Rapid Commun. Mass Spectrom..

[14] Khandelwal M (2003). Structural changes in silicon rubber peritoneal dialysis catheters in patients using mupirocin at the exit site. Int. J. Artif. Organs.

[15] Gardezi AI (2016). Erosion of the silicone peritoneal dialysis catheter with the use of gentamicin cream at the exit site. Adv. Perit. Dial..

[16] Kaneshiro N (2016). Rupture of subcutaneous peritoneal dialysis catheter by stretching exercise: A case report. Adv. Perit. Dial..

[17] Guiserix J (1997). Spontaneous rupture of peritoneal catheters. Nephron.

[18] Closkey GM, Zappacosta AR (1992). CAPD drainage failure due to Tenckhoff catheter fracture: A case report. Perit. Dial. Int..

